# Predicting Drug Release from 3D Printed Oral Medicines Based on the Surface Area to Volume Ratio of Tablet Geometry

**DOI:** 10.3390/pharmaceutics13091453

**Published:** 2021-09-11

**Authors:** Hellen Windolf, Rebecca Chamberlain, Julian Quodbach

**Affiliations:** Institute of Pharmaceutics and Biopharmaceutics, Heinrich Heine University, Universitätsstr. 1, 40225 Düsseldorf, Germany; hellen.windolf@hhu.de (H.W.); rebecca.chamberlain@hhu.de (R.C.)

**Keywords:** 3D printing, oral dosage form, drug dissolution, mean dissolution time, drug release prediction, personalized medicine

## Abstract

3D printing offers the advantage of being able to modify dosage form geometry, which can be exploited to modify release characteristics. In this study, we investigated the influence of the surface area to volume ratio (SA/V) to change and predict release profiles of 3D printed dosage forms. Geometries with varying SA/V and dosages were designed and printed, and drug dissolution was investigated. Three drug substances were used: pramipexole, levodopa (both BCS I) and praziquantel (BCS II). Two polymers were chosen as matrix formers: polyvinyl alcohol (water-soluble) and ethylene vinyl acetate (inert). Drug release was characterized using the mean dissolution time (MDT) and established equations that describe complete dissolution curves were applied. Predictions were validated with previously un-printed dosage forms. Based on an identified MDT-SA/V correlation, the MDT can be predicted with a deviation of ≤5 min for a given SA/V. Using correlations of fit parameters and SA/V, RMSEP values of 0.6–2.8% and 1.6–3.4% were obtained for the BCS I formulations and RMSEP values of 1.0–3.8% were obtained for the BCS II formulation, indicating accurate prediction over a wide range of dissolution profiles. With this approach, MDT and release profiles of dosage forms with a given SA/V can be precisely predicted without performing dissolution tests and vice versa, the required SA/V can be predicted for a desired release profile.

## 1. Introduction

Personalized pharmaceutical therapies are increasingly moving into focus to match the individual needs of patients [1,2,3]. There is a constantly improving understanding of the organism and metabolism of children and elderly patients, and it is becoming increasingly clear that drug substances may be metabolized differently compared to adults, especially if comorbidities are present. The efficacy of the active pharmaceutical ingredient (API) varies depending on age, body type, gender, health, and the individual metabolic state [4,5,6]. To adjust the dose of medicine for patients based on these dependencies, several approaches are available. Liquid or semi-solid preparations can be measured and dosed individually with the help of dosing aids, such as spoons, syringes, or cups. With solid dosage forms, it becomes more difficult to provide exact doses. Some tablets can be split into halves or quarters by hand or with the help of a tablet cutter, but these methods lead to inaccurate dosing [7,8]. Tablets with modified release especially demonstrate a major challenge if the release-determining factor is controlled by a functional coating, which loses its integrity when a tablet is split or mortared [9]. The requirements for dose variability and the constraining demand for an equivalent release profile despite the change in dose strength cannot be addressed industrially. The small batches needed for personalized medicine are not technically and economically feasible to manufacture on large-scale equipment. To provide patients with customized medicines, 3D printing to manufacture individualized medicine has been investigated [10,11,12,13,14]. Most commercial 3D printers are not only inexpensive, but also easy to operate and able to produce small batches for patients on demand. With individually manufactured dosage forms, it is possible to adapt the dosage of the API and the release kinetics of the API to the patient. In this way, a wide range of therapeutic regimens can be covered and side effects reduced, as the most effective dose with the least adverse effects can be administered precisely [15]. In the future, the production of these individual batches could be realized by community pharmacies, hospitals and regional manufacturing hubs.

In fused deposition modeling (FDM) 3D printing, intermediates containing the API, so-called filaments, are produced by hot-melt extrusion [3,16,17,18]. Filaments have to meet certain mechanical properties in order to enable the subsequent printing process [16,19,20]. The drug-loaded filament is fed into the print head, molten in the nozzle, and deposited on a heated print bed. Because of these two heat-intensive steps, FDM 3D printing is limited in terms of the APIs that can be used. The APIs should be thermostable and only decompose at high temperatures. Although it is possible to work with polymers that melt at low temperatures, this severely limits the choice of polymers [21]. The desired object is designed in advance using computer-aided design (CAD) software and converted via a slicer software into a machine language, the G-code. This G-code determines the temperature, movement, and the speed of the print head and print bed. The design of the object should consider the volume, which determines the dosage depending on the drug-load of the filament. There are two opportunities to influence the drug release properties of 3D printed objects: changing the composition of the filaments and adapting the surface area to volume (SA/V) ratio [22,23,24,25]. Since printing technologies allow one to manufacture a wide range of geometries without further development efforts, changing the SA/V ratio is the preferred option. It has been shown that the API release is faster, when the SA/V ratio is higher and slower when the ratio is smaller [22,23,26]. The absolute volume and the selected geometry were found to be negligible [23]. In order to reduce the elaborate production of filaments, there is a need to achieve different release profiles with only one filament.

To enable an individual dosage form with a defined dissolution profile, one must understand how the underlying formulation performs and according to which kinetic the API is released from the polymer matrix. Release profiles can be fitted using mathematical equations that consider the dissolution behaviour of the API and the polymer, as well as the diffusion pathways the API and the medium must overcome [27,28,29]. So far, various approaches have been used to try to predict the drug release from solid dosage forms [30,31,32,33,34,35,36,37,38,39,40,41]. Korte et al. investigated the approach of changing the dose of the 3D geometry via the percent of infill and predicted the release kinetics depending on the infill density [42]. Likewise, in another study, an infill in the form of honeycomb was modified, thereby also changing the drug release profile and predicting the resulting release profiles [43]. Artificial neural networks (ANN) are also being implemented to identify the influence of formulation and process parameters on the release behaviour and to improve predictions [28,34,44,45,46,47]. For example, Novák investigated the influence of varying infills and resulting tablet porosities on drug release using an ANN. This resulted in an in-silico design method of infill variations for 3D printed dosage geometries [48].

In this work, two different approaches were applied to predict dissolution characteristics and complete dissolution profiles of 3D printed geometries. This should ensure that individual batches can be produced in hospitals and community pharmacies without having to spend a lot of material, time and money on release testing. Firstly, the release behaviour of various geometries should be predicted via correlations between the SA/V ratio of the printed objects and the mean dissolution time (MDT) of the printed dosage forms. Secondly, complete release profiles should be predicted based on correlations between the SA/V ratio and suitable mathematical equations. Validation experiments should be performed in all cases to appraise the quality of the prediction approaches.

## 2. Materials and Methods

### 2.1. Materials

Formulation 1, referred to as the PVA-PDM formulation, consisted of 5% (*w*/*w*) pramipexole dihydrochloride monohydrate (PDM, Chr. Olesen, Denmark) as an API of the biopharmaceutics classification system (BCS) class I, declared as good water solubility (c_s_ ≥ 200 mg/mL) [49,50]. Mannitol (Parteck M^®^, Merck, Germany) was used as a plasticizer at 10% (*w*/*w*) content. Polyvinyl alcohol (84%, PVA, Parteck MXP^®^, Merck, Germany) was selected as a polymer. Formulation 2, referred to as the EVA-LD formulation, consisted of 10% (*w*/*w*) levodopa (Zhejiang Wild Wind Pharmaceutical, Dongyang, China), and was also a BCS class I API (c_s_ ≥ 12 mg/mL) [51]. As water soluble component, a vinylpyrrolidone-vinyl acetate copolymer (VP-VA) was used (39.5%, Kollidon VA 64^®^, BASF, Ludwigshafen, Germany) and 10% mannitol was added as a plasticizer. The matrix consisted of ethylene vinyl acetate (EVA) with a content of 18% vinyl acetate (39.5%, Escorene^®^ FL 01418, TER Chemicals, Hamburg, Germany). To improve flowability, 1% fumed silica (Aerosil^®^ 200 VV Pharma, Evonik, Germany) was added to both formulations. Formulation 3, referred to as the PVA-PZQ formulation, consisted of 5% (*w*/*w*) praziquantel (PZQ, donated from Bayer AG, Leverkusen, Germany) as an API of BCS class II (c_s_ = 0.4 mg/mL) [52,53,54], declared as poorly water-soluble. As a polymer basis, PVA was chosen with 95% content. All filament formulations were systematically developed to minimize diameter fluctuations of the filaments and to ensure highest printability with the available equipment. The criteria for the drug selection were heat stability and different classifications in the BCS. The melting point of PDM is also the decomposition point at 296–305 °C [20,55,56,57]. LD melts and decomposes at 260–330 °C [58]. PZQ has its melting point already at 140–143 °C but decomposes only at temperatures >400 °C [59,60]. The investigated dose ranges do not correspond to therapeutic dosages and result from the drug loading of the filament and the volume of the objects. The polymer matrix of the first formulation should be water soluble and generate prolonged drug release. PVA fulfils both criteria as the polymer forms a water-soluble hydrocolloid matrix [61]. To test the transferability of the predictive model to other formulations, an inert, non-swelling matrix, EVA, was chosen [62]. To improve the printability and hydrophilicity of the filament, VP-VA was added. PVA was again chosen for transferring the model to the BCS class II drug, for the extended drug release as well as better printability. Thus, variations resulting from the process could be eliminated and the differences clearly attributed to the API.

### 2.2. Methods

#### 2.2.1. Hot Melt Extrusion

The drug-containing filaments of formulation 1, 2 and 3 were produced via hot-melt extrusion with a co-rotating twin-screw extruder (Pharmalab HME 16, Thermo Fisher Scientific, Waltham, USA) using an in-house manufactured die with a diameter of 1.85 mm. The feed rate was set to 5 g/min and the screw speed to 30 rpm. The temperatures of the heating zones as well as the screw configurations are shown in Table 1. The filaments were hauled off with a winder (Model 846700, Brabender, Duisburg, Germany) at a speed of 1.8 m/min to the target diameter of 1.75 mm. The diameter was controlled using a laser-based diameter measurement module (Laser 2025 T, Sikora, Bremen, Germany).

#### 2.2.2. 3D Printing of Tablets

The drug-loaded filaments were printed on a FDM 3D printer (Prusa i3 Mk3, Prusa Research, Prague, Czech Republic) to oral dosage forms in various geometries. The geometries were designed with Fusion 360^®^ (Autodesk, San Rafael, CA, USA) and sliced in Simplify3D^®^ (Simplify3D, Cincinnati, OH, USA) to obtain the desired G-code. The print temperature for the PVA-PDM filaments was set to 185 °C, the bed temperature to 60 °C and the printing speed was 20 mm/s. The printing temperature had to be increased for the EVA-LD formulation, as the filament was very flexible and could not be transported reliably through the nozzle by the conveying wheels in the print head at lower temperatures, as the filament would otherwise wrap around the wheels. To ensure a constant filament-flow through the nozzle, the temperature of the nozzle was set to 220 °C and the printing speed was reduced to 10 mm/s. For the PVA-PZQ formulation, a nozzle temperature of 185 °C could again be used, but the bed temperature had to be increased to 90 °C because the printed objects adhered poorly to the print bed and detached from the bed more often. Therefore, a printing speed of 10 mm/s was also selected here. To obtain high print accuracy, the layer height was set to 0.1 mm and the extrusion width to 0.4 mm using a nozzle with a diameter of 0.4 mm. The infill percentage of the concentric infill was set to 100%.

#### 2.2.3. Dissolution Test

According to Ph. Eur. monographs 2.9.3 and 5.17.1 [63,64], release studies (*n* ≥ 3) were performed with the basket method (Method 1) in a dissolution tester (DT 726, Erweka, Langen, Germany). The baskets were 3D printed from water insoluble polylactide acid (PLA). They had to be adapted for printed tablets, since the mesh size of the regular Ph. Eur. baskets is small (0.36–0.44 mm) and the baskets were clogged by the swollen polymer of the PVA formulation. This affected the hydrodynamic medium flow around the printed oral dosage forms. The self-printed baskets have the same outer dimensions as the Ph. Eur. baskets, except that the mesh width was changed to 3 mm. The dissolution test for PDM containing tablets was performed in 500 mL of degassed 0.1 N hydrochloric acid at pH 1.2 under sink conditions (c_s_ ≥ 200 mg/mL [50]; maximum concentration 0.08 mg/mL) and stirred at 50 rpm at a temperature of 37 ± 0.5 °C. The released API was measured using an UV-Vis spectral photometer (UV-1800 Shimadzu, Japan) at a wavelength of 263 nm. Dissolution testing of the levodopa containing geometries was performed in 1000 mL of degassed 0.1 N hydrochloric acid at pH 1.2 under sink conditions (c_s_ ≥ 12 mg/mL [51]; maximum concentration 0.05 mg/mL) and stirred at 50 rpm at a temperature of 37 ± 0.5 °C. The API release was recorded with the same UV-Vis spectral photometer at a wavelength of 280 nm. The dissolution tests with PZQ were performed in 750 mL of degassed 0.1 N hydrochloric acid at pH 1.2 for the first 120 min and then transferred to 1000 mL phosphate buffer pH 6.8 with 250 mL of degassed 0.2 N tri-sodium phosphate dodecahydrate solution. The temperature was adjusted to 37 ± 0.5 °C. The drug release was measured at a wavelength of 210 nm. The tests were performed under sink conditions (c_s_ = 0.4 mg/mL [54]; maximum concentration: 0.03 mg/mL). Samples were taken every 5 min for the first 30 min, then every 10 min for the next 90 min, followed by sampling in 20 min intervals. After a release time of 240 min, samples were taken every 30 min.

#### 2.2.4. Mathematical Description

##### Release Modeling

The mean dissolution time (MDT) is used as a characteristic value that describes the drug release and was calculated according to Equation (1). It is expressed in units of time, usually in minutes, and indicates how long an API molecule is retained in a dosage form on average during dissolution [27,65].

(1) $$\mathrm{MDT}=\frac{\mathrm{ABC}}{c_{\infty }}=\frac{\sum_{i=0}^{\infty }\left[\left(c_{i+1}-c_{i}\right)*\frac{\left(t_{i}+t_{i+1}\right)}{2}\right]}{c_{\infty }}$$

ABC stands for the area between the curves and is calculated via the trapezoidal equation with c as the concentration of the API released over time t and $c_{\infty }$ as the initial drug load of the dosage form. Values up to 100% of the release curve were used for the calculation since the ABC does not change afterwards.

Various mathematical models were tested to fit the complete release profiles for prediction. First, the Korsmeyer–Peppas equation, or power law, was applied (Equation (2)) [27,66].

(2) $$\frac{M_{t}}{M_{\infty }}={k*t}^{n}$$

The released amount of the API over time t is described by the term $M_{t}$. The total amount of the API in the dosage form at the beginning of the release process is represented by $M_{\infty }$. The constant k describes the structural and geometrical characteristics of the dosage form (cylinder, sphere, film), also known as the reaction rate constant or release velocity constant. The diffusional constant n describes the underlying drug release mechanism. Only the first 60% of the release curve should be used for the calculation. The model can be used to analyse the underlying release properties of the system, when the mechanism is unknown, or more than one release mechanism is involved. It should be taken into account that the equation requires some properties of the matrix to be valid: the drug in the matrix must be distributed homogeneously, show unidirectional diffusion, and during dissolution, sink conditions should be maintained. Depending on the value of n, the release behaviour can be categorized into Fickian Diffusion, anomalous transport, or case-II transport (see Table 2) [29,66].

The second equation used for the description of the release profile was the Peppas Sahlin equation (Equation (3)) [29].

(3) $$\frac{M_{t}}{M_{\infty }}=k_{1}\times t^{n}+k_{2}\times t^{2n}$$

Using this equation, it is possible to describe the anomalous drug release process: the first term describes the Fickian diffusion and the second the Case-II relaxational contribution. The exponent n is, as in the Korsmeyer–Peppas equation, the diffusion exponent for any geometrical shape. The constants $k_{1}$, $k_{2}$ describe the kinetics. The equation refers only to the anomalous release of the API [67].

The drug release from an inert matrix is often described using the Higuchi equation (Equation (4)). The Higuchi equation describes the release of a suspended drug from an insoluble matrix via diffusion and is also called square-root-of-time kinetic. This equation was initially intended for ointments but can also be used for solid dosage forms [68,69].

(4) $$Q=\sqrt{D\times \left(2\times c_{0}-c_{s}\right)\times c_{s}\times t}$$

This calculation is used to calculate Q, the amount of API released at time t per unit area. It is composed of D, the diffusion coefficient of the API in the matrix, $c_{0}$, the initial dose in the dosage form and $c_{s}$, the saturation concentration of the API. The formula considers that the diffusion distance covered by the API does not remain constant but increases steadily during dissolution. The API concentration in the matrix decreases towards the medium since this is where the API is released first. Due to the growth of the diffusion distance, the concentration gradient decreases and, accordingly, the release rate. The Higuchi equation can be applied to suspension ointments and planar matrices (films) in which the API is dispersed as the equation describes a one-dimensional diffusion under sink conditions. The matrix should be inert and not undergo any change in structure, not even swelling, so that the diffusion coefficient remains constant. In addition, the drug concentration in the dosage form must be higher than the saturation solubility c_s_. As soon as these conditions are no longer given, the equation is invalid, since the API is then no longer dispersed but dissolved. The application of this equation is limited to 60% of the released API [70].

Lapidus and Lordi modified the Higuchi equation for swellable hydroxypropyl methylcellulose (HPMC) tablets (Equation (5)) [22,71,72].

(5) $$\frac{M_{t}}{M_{0}}=2\times \left(\frac{\mathrm{SA}}{V}\right)\times {\left(\frac{D\times t}{\pi }\right)}^{0.5}$$

In this equation, $M_{t}$ represents the released API at time t and $M_{0}$ indicates the initial amount of the API in the tablet. The formula directly includes the SA/V ratio and the diffusion coefficient D of the API in the matrix. 

For eroding tablets, the release can be described by the Hixson Crowell equation (Equation (6)). The formula reflects the size decrease of the dosage form. It assumes that the surface of the form decreases proportionally over time and the geometrical form remains constant.

(6) $$\sqrt[{\;3}]{W_{0}}=\sqrt[{3}]{W_{i}}+k\times t$$

$W_{0}$ reflects the initial amount of API and $W_{i}$ is the remaining API content over time t. The constant k is the constant of incorporation, which describes the surface area and volume relation of the dosage form. For the Hixson Crowell model, the release described is limited by dissolution velocity and not by diffusion of the API through the polymer matrix. Throughout dissolution, it is assumed that the surface decreases as layers detach over time [27,73,74].

Furthermore, a release of an eroding matrix can be described by the Hopfenberg equation (Equation (7)). The Hopfenberg equation is used to describe the drug release from different geometries (plates, spheres, and cylinders). The assumption is that drug release occurs via erosion and is not affected by diffusion.

(7) $$\frac{M_{t}}{M_{\infty }}=1-{\left[1-\frac{k_{0}\times t}{c_{o}\times a_{0}}\right]}^{n}$$

The term $\frac{M_{t}}{M_{\infty }}$ describes the release of the initial concentration $c_{o}$ of the system over time t with $M_{t}$ as API amount at the observed point of time and $M_{\infty }$ as the total amount of API solute of the dissolved tablet at infinite time. The erosion constant is described by $k_{0}$. The variable $a_{0}$ presents the radius of the selected geometry (sphere, cylinder, plate). The factor n represents the shape of the dosage form: n = 1 for a plate, n = 2 for a cylinder and n = 3 for a sphere. The limiting factor of this model is erosion of the matrix, which is the rate-limiting step of drug release and assumes that time-dependent diffusion resistances inside or outside the eroding matrix have no effect on it [27,75,76].

The Weibull equation (Equation (8)) can be used to describe release curve profiles, regardless of the underlying physical mechanisms (diffusion or erosion).

(8) $$c_{t}=1-e^{\left(\frac{-{\left(t-T_{i}\right)}^{b}}{a}\right)}$$

The value of $c_{t}$ is the concentration of the API at timepoint t. $T_{i}$ represents the lag-time before the release of API starts. If there is no delayed release, $T_{i}$ becomes zero. The parameter b describes the shape of the curve (exponential b = 1; sigmoid b > 1 or parabolic b < 1). The scale parameter a characterizes the time scale of the dissolution. As this equation is an empirical model without any kinetic basis, it does not provide any information about the underlying release properties, and only describes the curve profile. [27,77,78,79].

##### Prediction

In order to be able to evaluate how accurate the prediction of the release curves is, the root mean square error of prediction (RMSEP) was calculated (Equation (9)) [42,80].

(9) $$\mathrm{RMSEP}=\sqrt{\frac{\sum_{i=1}^{n}{\left(y_{i}-\;\hat{y}\right)}^{2}}{n}}$$

The RMSEP value describes the differences between the predicted and the measured values. The factor $y_{i}$ represents the experimental values and $\hat{y}$ represents the prediction values for every point of time of the release curve. The number of samples is indicated by n.

##### Comparison of the Dissolution Profiles

To compare the release curves, the similarity factor was used. This is approved by the FDA. The calculation was performed with Equation (10) [27,81,82].

(10) $$f_{2}=50\times \log\left\{{\left[1+\frac{1}{n}\overset{n}{\underset{t=1}{\sum }}{\left(R_{t}-T_{t}\right)}^{2}\right]}^{-0.5}\times 100\right\}$$

In this equation, $R_{t}$ and $T_{t}$ stand for the mean released amounts of the API in % at time point t of the reference and the test product and n for the number of time points. A minimum of 12 measurement points was used as the mean to determine the $f_{2}$ value. The $f_{2}$ value is sensitive to the number of measurement points. For this reason, only one measurement point was included in the calculation after 85% released API. A $f_{2}$ value around 100 is desired, which indicates that the curves are identical. A value of 50 or more is accepted, which indicates that the values differ by max. 10%. Values below 50 indicate that the curves can no longer be considered similar.

#### 2.2.5. Characterization of the Printed Tablets

To evaluate the precision of the printing and to see whether the designed SA/V ratio was also adhered to in the printed geometries, the printed tablets were measured with a caliper (CD-15CPXR, Mitutoyo, Kawasaki, Japan). The weight of the tablets was determined on a balance (Type 1702, Sartorius, Göttingen, Germany), and the tensile strength of the tablets was determined (*n* = 10) with the crushing force tester (TBH210, Erweka, Langen, Germany). 

## 3. Results

### 3.1. Characterization of the Printed Tablets

To ensure the printed tablets corresponded to the designed SA/V ratio, they were characterized (Supplementary Material, Tables S1–S7). The printing precision is considered very good for all three formulations and the deviations within a batch are very low, which indicates a good reproducibility. The largest weight variations were found for the PZQ-PVA formulation. The larger and heavier the printed tablet, the higher the variations in weight. In contrast, the API content is very similar, so the variations are small. These data suggest a good extrusion process with a homogeneous API distribution, as well as a robust printing process with small variations in precision. This leads to small variations in mass, which in turn leads to small variations in content. The printed PVA tablets have a high internal hardness, which means that they cannot be broken in the crushing force tester. The EVA tablets are very flexible, so that they also do not break in the crushing force tester but deform. 

### 3.2. Drug Release from Dosage Forms with Defined SA/V Ratios

**Figure 1:**
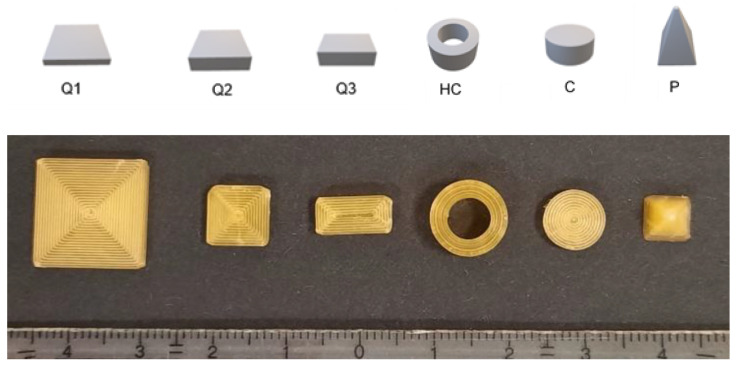
Designed geometries (from left to right): cube 1 (Q1), cube 2 (Q2), cube 3 (Q3), hollow cylinder (HC), cylinder (C) and pyramid (P). Upper image: CAD-Design, lower image: printed geometries of SA/V ratio 1.5 mm^−1^.

For initial release studies, various geometries of constant SA/V ratios with different doses were printed with the PVA-PDM formulation (Figure 1). The SA/V ratios were 1, 1.5, and 2 mm^−1^, based on changes of the dimensions (Table 3). By keeping the SA/V ratio constant, the dose was varied according to the volume of the objects.

**Figure 2:**
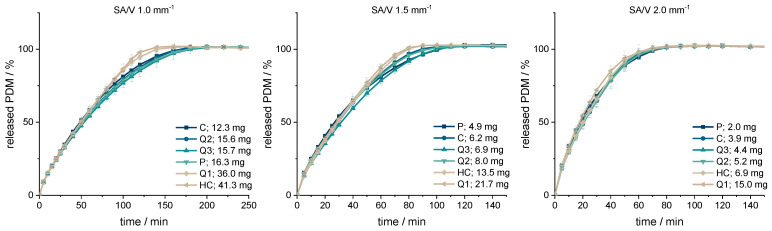
PDM-release curves of the geometries printed with SA/V ratios of 1, 1.5, 2 mm^−1^ (*n* = 6; x ± s).

The dissolution data (Figure 2) revealed that dose and size did not influence the relative drug release but only the SA/V ratio, which was expected. The PVA matrix forms a hydrocolloid structure that swells first and slowly dissolves over time.

The smaller the SA/V ratio was, the longer it took until 100% of the API was released as the diffusion pathways are longer and vice versa. The geometries with a SA/V ratio of 1 mm^−1^ released 80% of the API in 100 min, the geometries with a SA/V ratio of 1.5 mm^−1^ released 80% in 60 min and with a SA/V ratio of 2 mm^−1^, 80% API was released in 45 min. The dosages varied by a factor of 7.5 for the SA/V ratio of 2 mm^−1^, by a factor of 4.5 for the SA/V ratio of 1.5 mm^−1^ and by a factor of 3.4 for the ratio of 1 mm^− 1^.

The respective MDTs were calculated using Equation (1) and are listed in Table 3. Geometries with the same SA/V ratio have very similar MDTs that match the observed trend of the dissolution data. The MDT of geometries printed with a SA/V ratio of 1 mm^−1^ was 61 ± 3 min, with a SA/V ratio of 1.5 mm^−1^ 35 ± 2 min and with a SA/V ratio of 2 mm^−1^ 25 ± 1 min across all designs. The similarity of the curves was evaluated with the similarity factor (Equation (10)). The cylinder was used as a reference for each SA/V group. The f_2_-values are above 50 in each group and show that the curves are similar to each other.

### 3.3. Correlation between MDT and SA/V Ratio

**Figure 3:**
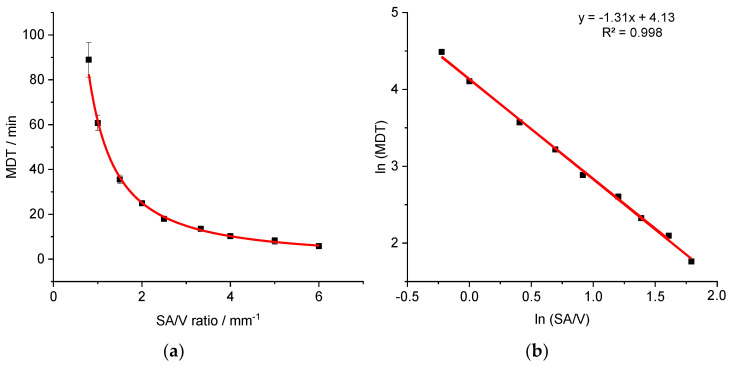
Correlation of MDT and SA/V ratio for the PVA-PDM formulation (**a**) and linearized version (**b**) (*n* ≥ 3; x ± s).

Based on this data, a correlation between the SA/V ratio and the MDT was hypothesized. For a better understanding of the correlation between these two features, geometries with additional SA/V ratios were printed and the API release tested with the PVA-PDM formulation. For this, a SA/V ratio of 0.8 mm^−1^ was chosen as minimum and 6.0 mm^−1^ as maximum. The obtained data is shown in Figure 3a.

The correlation between the variables SA/V and MDT results in a curve ($\mathrm{MDT}=62.5\times {\left(\frac{\mathrm{SA}}{V}\right)}^{-1.3}$), which could be linearized by log-transformation of both axes (R^2^: 0.9977). The linear equation of the regression was used to predict the MDT of 3D printed tablets with different SA/V ratios. The predictions were made exemplarily for four different SA/V ratios not used for the regression model and compared with the experimentally determined MDT of the corresponding printed geometries (Table 4).

The predictions are very similar to the measured MDTs. The deviations are less than 5 min, which is acceptable with respect to small variations in the measured release curves. With this assessment, it is possible to categorize the SA/V ratios in terms of their release profiles.

**Figure 4:**
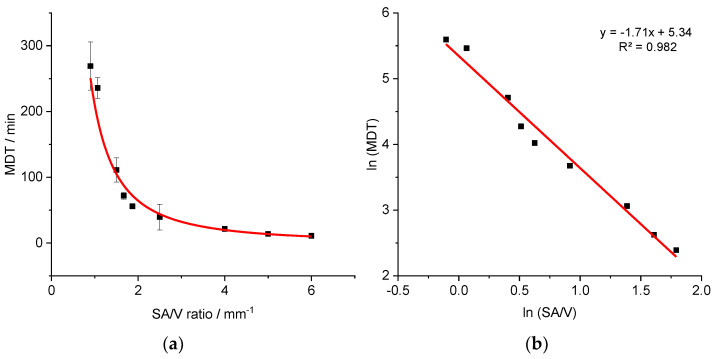
Correlation of MDT and SA/V ratio for the EVA-LD formulation (**a**) and linearized version (**b**) (*n* ≥ 3; x ± s).

Similar experiments were conducted for the EVA-LD formulation to assess the validity of the approach. Geometries with variable dosages and SA/V ratios of 0.9–6.0 mm^−1^ were printed, and the MDT was calculated from the obtained dissolution data (Figure 4).

A similar relationship was obtained as described earlier for the PVA-PDM formulation. The resulting curve ($\mathrm{MDT}=209.43\times {\left(\frac{\mathrm{SA}}{V}\right)}^{-1.71}$) was linearized again via log-transformation of both axes (R^2^: 0.9819).

Additional geometries with defined SA/V ratios were printed and the MDTs resulting from the release curves were calculated. The measured and predicted MDTs are shown in Table 5.

Again, predicted MDTs describe the obtained data well. The variations between the predicted values to the experimentally determined values are similar to the variations from the PVA-PDM formulation.

**Figure 5:**
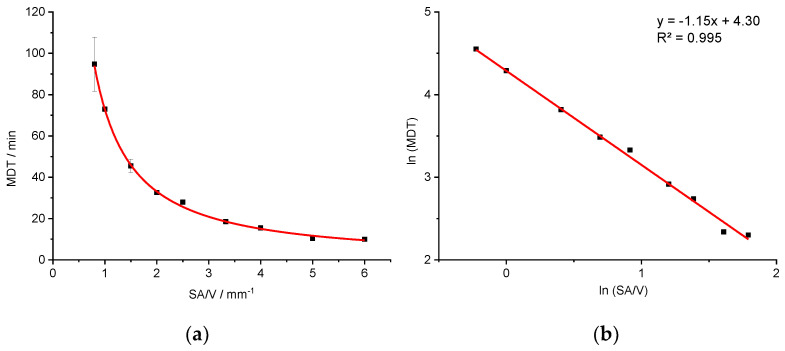
Correlation of MDT and SA/V ratio for the PVA-PZQ formulation (**a**) and linearized version (**b**) (*n* ≥ 3; x ± s).

The approach for predicting MDT was also tested for BCS class II using the PVA-PZQ formulation (Figure 5).

Likewise, a relationship between the SA/V ratio and the MDT ($\mathrm{MDT}=73.487\times {\left(\frac{\mathrm{SA}}{V}\right)}^{-1.145}$) is evident. A linearization is again obtained by a log-transformation of both axes (R^2^: 0.9953).

Additional geometries with defined SA/V ratios were printed (not used for the regression model) and the MDTs resulting from the release curves were calculated. The measured and predicted MDTs are shown in Table 6.

Again, predicted MDTs describe the obtained data precisely. The variations between the predicted values and the experimentally determined values are similar to the variations from the PVA-PDM or EVA-LD formulation.

In the inert EVA matrix, which does not swell nor erode, the API can only be dissolved and transported by diffusion through the inert matrix into the dissolution medium. The API release is more prolonged compared to the PVA matrix. Considering geometries with a SA/V ratio of 1.5 mm^−1^, the drug was released from the PVA-PDM matrix with a MDT of 35.58 ± 1.87 min, from the PVA-PZQ matrix with a MDT of 45.49 ± 3.27 min and from the EVA-LD matrix with a MDT of 111.07 ± 18.37 min. Despite the different behaviour of the matrix systems, the solubility properties of the APIs and the resulting differences in MDT, a correlation of the SA/V ratio and the MDT can be established for all three tested formulations. With the help of this correlation, the release time of an existing geometry with a given SA/V ratio can be predicted without having to waste material and perform dissolution tests. Similarly, the required SA/V ratio can be predicted for a desired MDT and the corresponding geometry can be designed and printed on this basis. 

If it can be demonstrated that a linear relationship exists between ln(SA/V) and ln(MDT), a simple regression model can be generated using a two-point calibration with dissolution data only from geometries with a low and a high SA/V ratio. This would save further time and production costs and valid predictions could still be made.

### 3.4. Modeling and Prediction of Release Profiles

To predict the complete release curve and not only the MDT, different mathematical models were used to fit the data (Equations (2)–(8)) and the R^2^ values of the fits were compared to evaluate the fit quality (Table 7). According to the Korsmeyer–Peppas model (KMP), the drug release from the PVA formulations follows an anomalous transport (diffusion constant *n* = 0.7 (PDM), *n* = 0.8 (PZQ), Table 2). Since anomalous transport can be described with different models (Equations (3) and (5)–(8)), only these equations were used to fit the data of the PVA formulations. For the EVA-LD formulation, *n* = 0.55 was obtained. This indicates a release according to square-root-of-t kinetic. This profile is often described with the Higuchi equation (Equation (4)). Additionally, the generally applicable Weibull equation (Equation (8)) as well as the Peppas Sahlin equation (Equation (3)) were used to fit the release profiles of the EVA formulation. 

**Figure 6:**
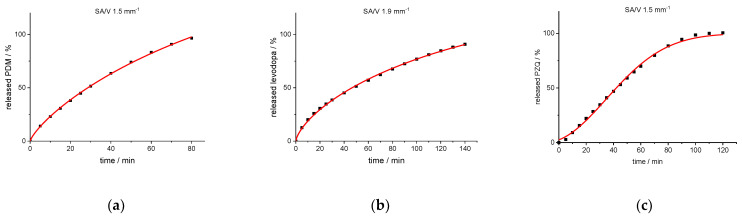
Experimental release data points (black cuboids) and predicted release curve (red line) calculated with Peppas Sahlin (PVA-PDM formulation (**a**); EVA-LD formulation (**b**)) and Weibull function (PVA-PZQ formulation (**c**)).

For both BCS class I API formulations, the Peppas Sahlin equation provides the best fit. In order to also include enough data points for fast release dosage forms, the curves were fitted up to an API release of 98%. For the formulation with BCS class II API, the Weibull function provides the best fit (Figure 6).

**Figure 7:**
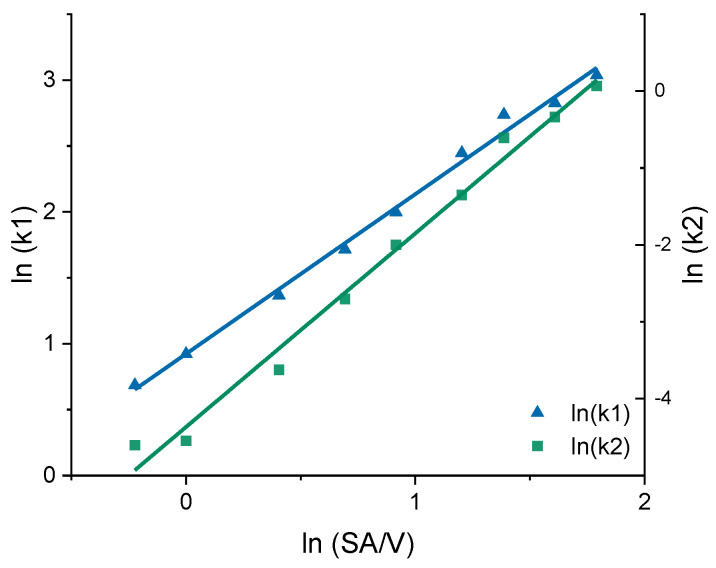
Correlation of the constants k_1_ and k_2_ of the Peppas Sahlin equation with the SA/V ratio for the PVA-PDM formulation.

In earlier works, the slope of the Higuchi plot, the rate constant of dissolution and the constant k of the Korsmeyer–Peppas equation were already related to the particle size and the SA/V ratio of tablets [22,83,84]. In this study, all dissolution curves of BCS class I APIs were fitted with the Peppas Sahlin equation, as it provided the best fits and the constants k_1_ and k_2_ were determined (Table 8). For the PVA-PDM formulation, an average diffusion exponent of *n* = 0.79 was obtained, and for the EVA-LD formulation, an average diffusion exponent of *n* = 0.66 was obtained. The constants k_1_ and k_2_ were plotted in a graph as a function of the SA/V ratio. A linear correlation between k and the SA/V ratio was obtained by a log-transformation of both axes (Figure 7).

**Figure 8:**
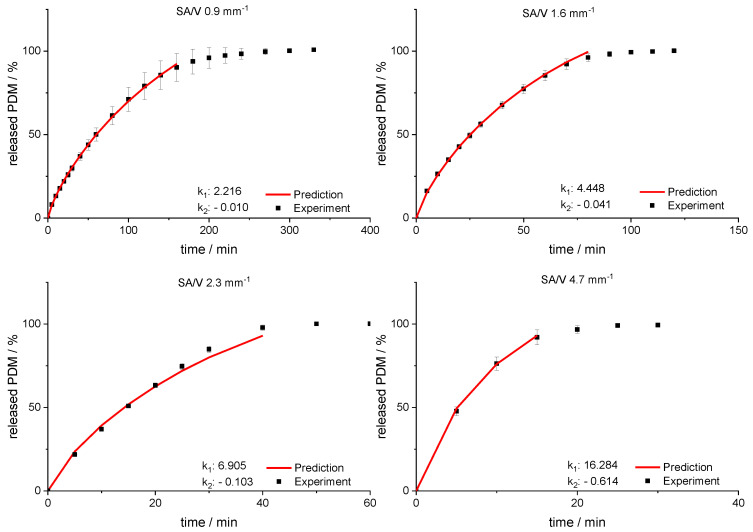
Predicted PDM release profiles vs. experimental results for SA/V ratio 0.9 mm^−1^, 1.6 mm^−1^, 2.3 mm^−1^ and 4.67 mm^− 1^ of the PVA-PDM formulation.

Using this relation and the resulting linear regression, k_1_ and k_2_ can be determined for arbitrarily selected SA/V ratios. If these calculated values are inserted into the Peppas Sahlin equation with the diffusion exponent *n* = 0.79, a concentration curve of the released API can be calculated for the specific time points. These calculations were performed exemplarily for the four different SA/V ratios not used for the model creation. The graphs comparing the experimental data points with the prediction curve are shown in Figure 8.

The RMSEPs were calculated (Equation (9)) for the predictions and experimental results. The best predictive power was observed for a SA/V ratio of 1.6 mm^−1^ with a RMSEP of 0.56% and 0.9 mm^−1^ with a RMSEP of 0.74%. For the SA/V ratio of 2.3 mm^−1^, the prediction above 70% API release underestimated the experimental data points, which results in a RMSEP of 2.85%. If only the release data points up to 70% released API are included, the RMSEP was 1.70%. Predicting the SA/V ratio of 4.7 mm^−1^ resulted in a RMSEP of 3.41%. If the prediction is only made up to 70% API release, the RMSEP is 1.18%, but only three time points are compared due to the quick drug release. Apparently, the predictions represent the experimental values better for smaller SA/V ratios because multiple time points can be considered and therefore the curves are not as sensitive to changes in k values. Nevertheless, the prediction approximated the experimental release curves well, especially in the early phase of drug release.

**Figure 9:**
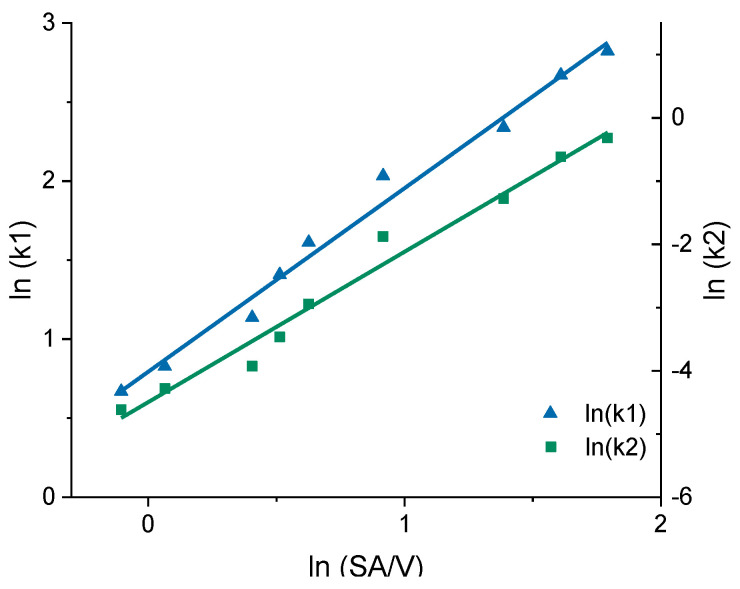
Correlation of the constants k_1_ and k_2_ of the Peppas Sahlin equation with the SA/V ratio for the EVA-LD formulation.

**Figure 10:**
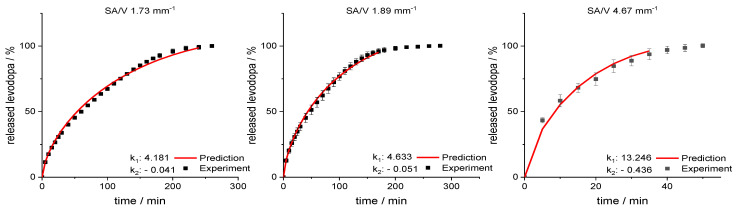
Predicted levodopa release vs. experimental levodopa release for SA/V ratios of 1.73 mm^−1^, 1.89 mm^−1^ and 4.67 mm^−1^ of the EVA-LD formulation.

The prediction approach was tested for the EVA-LD formulation, respectively. Again, the constants k_1_ and k_2_ were plotted in a graph as a function of the SA/V ratio. A linear correlation between k and the SA/V ratio was obtained by a log-transformation of both values (Figure 9). Using this relation and the resulting linear regression, k_1_ and k_2_ can be determined for arbitrarily selected SA/V ratios. These calculations were exemplarily for the three different SA/V ratios, the MDT was already predicted in Section 3.2. The resulting graphs comparing the experimental data points with the prediction curve are shown in Figure 10.

For the SA/V ratio of 1.89 mm^−1^, the RMSEP value was 1.60% and for the SA/V ratio of 1.73 mm^−1^, the RMSEP value was 1.95%. The RMSEP for the SA/V ratio 4.67 mm^−1^ results in a value of 3.42%. Again, the experimental data of the smaller SA//V ratios can be predicted better by the model, nevertheless, the prediction of higher SA/V ratios is still close to experimental data. The data implies that the developed prediction model also works for this formulation with an inert matrix. 

**Figure 11:**
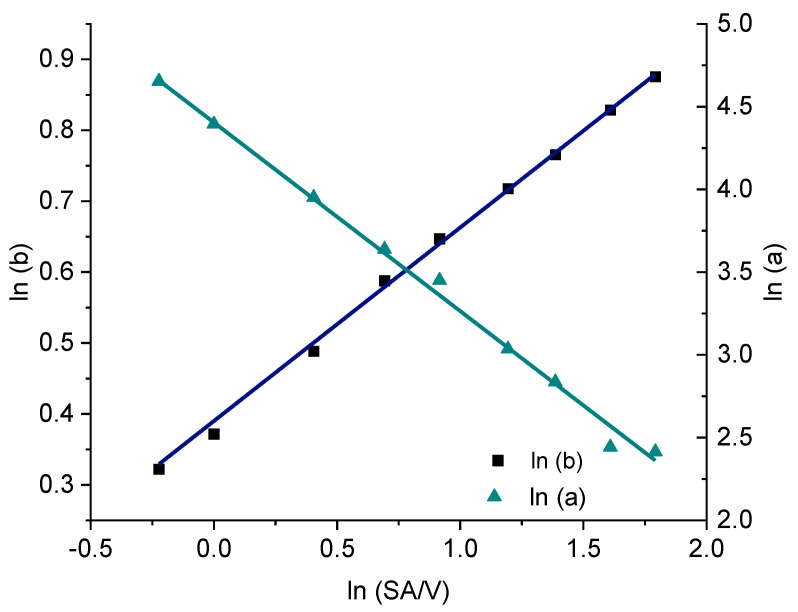
Correlation of the constants a and b of the Weibull function with the SA/V ratio for the PVA-PZQ formulation.

**Figure 12:**
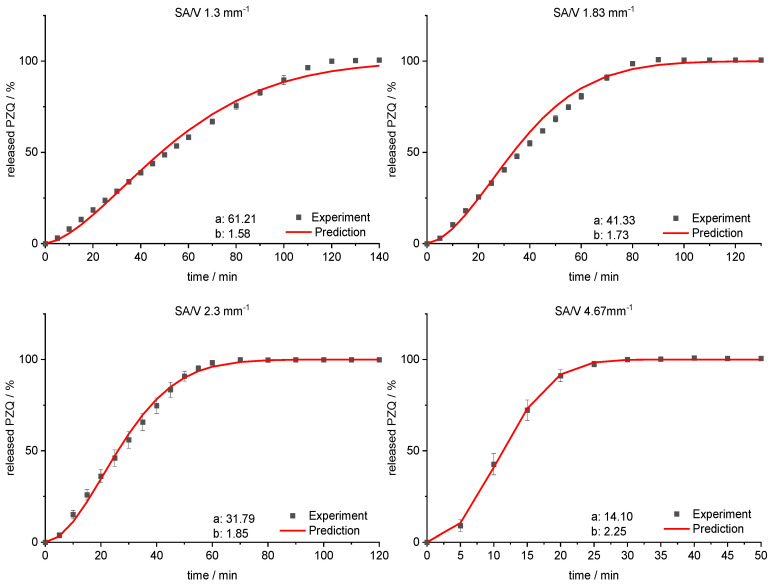
Predicted PZQ release vs. experimental PZQ release for SA/V ratios of 1.3 mm^−1^, 1.83 mm^−1^, 2.3 mm^−1^ and 4.67 mm^−1^ of the PVA-PZQ formulation.

For the PVA-PZQ formulation, the Weibull function provides the best fit (Table 7). Therefore, the approach for predicting the dissolution curves was tested for this equation. For the given SA/V ratio, the variables a and b were calculated (Table 9) and plotted with log-transformed axes (Figure 11). The resulting linear equations were used to calculate the required variables for the SA/V ratios, whose curves were to be predicted. The comparison of the predicted and experimentally determined release profiles is shown in Figure 12.

The sigmoidal curve is described with the Weibull function. Initially, the amount of API released is somewhat underestimated, but as dissolution progresses, the mean value is predicted well. For the curves of lower SA/V ratios, the course of the release curve is overestimated from 55–80% released API and a stronger S-curve course is predicted. With increasing SA/V ratio, however, the Weibull curve predicts the release profile better. The RMSEP for the SA/V ratio of 1.3 mm^−1^ is 2.5%, for 1.83 mm^−1^ 3.6%, for 2.3 mm^−1^ 2.1% and for 4.67 mm^−1^ 1.0%. The predicted value of the curve deviates from the experiment by 2.3% on average, which can be considered acceptable.

With this approach, it is possible to extend the prediction of the MDT as described in Section 3.2 and predict the release profile of different APIs of various BCS classes for a given SA/V ratio. As shown with the different formulations and APIs, this method can potentially be applied for various matrices and drug substances. This approach enables more accurate predictions to be made for the release behaviour of 3D printed dosage forms.

## 4. Discussion

A correlation between MDT and SA/V ratio was found in these experiments and used for a prediction of drug release profiles with three different formulations. With the help of this approach, the MDT of printed dosage forms with known SA/V ratios can be easily predicted and it is possible to categorize the SA/V ratios in terms of their release. MDT predictions are quite accurate for all three formulations, varying from experiment mostly by ≤5 min, which is acceptable. The largest variations are found for the EVA formulation, which may be due to the more difficult printing conditions. Due to the high flexibility of the filaments, the constant transport of the filament through the nozzle is not always given, resulting in less accurate printing of the tablets. In addition, the release of the API is longer than in the PVA formulations, resulting in larger standard deviations. Since inert matrices often lead to prolonged release kinetics and, therefore, require time consuming experiments, the identified relationship between MDT and SA/V ratio represents a helpful tool for dosage form design. Accurate predictions of release profiles of BCS class I APIs could be made using the Peppas Sahlin equation. This equation was used to describe the dissolution curves of two different matrix systems with different release characteristics. The constants of the equation were found to change in a predictable manner with the SA/V ratio. For both formulations, precise predictions of release profile could be made, with an average variation of 0.6–3% of API released per time point. Predictions for smaller SA/V were mostly more accurate than those for larger SA/V, which may be due to the number of time points included, making the curves more sensitive to changes in the constants. In the PVA formulation with the BCS class II compound, the Weibull function was found to be most suitable to describe the drug release (R^2^: 0.999). Again, the linear relationship between the logarithmised constants and the SA/V ratio was used to calculate dissolution predictions. With an average deviation of 2% from the predicted to the experimentally determined release profile, it can be stated that dissolution curves of dosage forms with a poorly water-soluble API can also be modeled.

Future studies should include testing more formulations and drug substances to investigate the validity of this approach for other drugs and polymers, and expanding the database, as well as investigating the use of ANNs for these kinds of dosage forms. In addition, it should be tested whether the prediction model is also transferable to tablets or dosage forms manufactured by other 3D printing processes, e.g., by powder bed 3D printing or semi-solid 3D printing with different materials and porosity.

## 5. Conclusions

We demonstrated that the relationships between constants of established equations and the SA/V ratio can be exploited to predict complete dissolution curves for specific SA/V ratios. The validity of this approach was shown for water-soluble as well as poorly water-soluble drug substances and erodible and inert polymers. It might be possible to establish a dataset for polymer matrices with various APIs of different BCS classes and directly calculate the release curves of dosage forms with a known SA/V ratio. Such an approach would save additional costs and time-consuming dissolution studies of individual geometries and matrix systems, which is especially important for community pharmacies and hospitals. Future patient care with 3D printed oral dosage forms could be: 1. the optimal drug release for a patient is determined, 2. the appropriate formulation and SA/V ratio are selected, 3. a suitable geometry is 3D printed with the required dose of the API. Therefore, with an established matrix system, not every drug release of oral dosage forms would need to be tested in dissolution studies but could be mathematically predicted. As a result, the dissolution rate could be individualized in decentralized settings, an approach that has not been easily adaptable before.

## Figures and Tables

**Table 1:** Extrusion settings (temperature profile and screw configuration) for the used formulations.

Temperature Profile in Zone 2–10/°C									
Zone/-	2	3	4	5	6	7	8	9	10
PVA-PDM formulation/°C	30	100	180	180	180	180	180	195	195
PVA-PZQ formulation/°C	21	31	78	180	180	180	180	180	190
EVA-LD formulation/°C	25	28	78	130	140	155	155	120	100
**Screw Configuration (Die–Gear)**									
PVA/EVA formulation	die-10 CE 1 L/D-KZ: 5 × 60°-3 × 30°-5 CE 1 L/D-KZ: 4 × 90°-5 × 60°-3 × 30°-10 CE 1 L/D-2 CE 3/2 L/D-gear								
CE = conveying element, KZ = kneading zone									

**Table 2:** Characterization of the diffusion exponent *n* depending on the dosage form geometry.

*n*			
Thin Film	Cylinder	Sphere	Drug Release Mechanism
0.50	0.45	0.43	Fickian diffusion
0.50 < *n* < 1.00	0.45 < *n* < 0.89	0.43 < *n* < 0.85	Anomalous transport
1.00	0.89	0.85	Case-II transport

**Table 3:** Characteristics of the printed geometries including the calculated MDT of the PVA-PDM formulation.

SA/V 1 mm^−1^						
Form	SA/mm^2^	V/mm^3^	SA/V/mm^−1^	API/mg	MDT/min	f_2_ Value
Q1	606.00	585.00	1.00	35.97	56.95	77.51
Q2	256.00	256.00	1.00	15.60	62.91	87.92
Q3	250.00	250.00	1.00	15.66	65.65	73.88
HC	667.59	667.59	1.00	41.34	56.84	71.87
C	201.06	201.06	1.00	12.29	60.67	Reference
P	273.05	265.97	1.03	16.25	64.30	82.89
**SA/V 1.5 mm^−1^**						
**Form**	**SA/mm^2^**	**V/mm^3^**	**SA/V/mm^−1^**	**API/mg**	**MDT/min**	**f_2_ Value**
Q1	546.00	360.00	1.52	21.74	32.81	78.20
Q2	192.00	128.00	1.50	8.00	35.67	92.54
Q3	166.00	110.00	1.51	6.92	38.07	73.19
HC	301.59	201.06	1.50	13.54	33.83	90.82
C	150.80	100.53	1.50	6.19	34.80	Reference
P	121.92	80.10	1.52	4.85	36.92	75.40
**SA/V 2 mm^−1^**						
**Form**	**SA/mm^2^**	**V/mm^3^**	**SA/V/mm^−1^**	**API/mg**	**MDT/min**	**f_2_ Value**
Q1	516.00	247.50	2.08	15.04	22.98	66.45
Q2	169.60	83.20	2.04	5.21	25.70	96.55
Q3	142.00	70.00	2.03	4.43	24.90	91.95
HC	201.06	100.50	2.00	6.87	25.02	92.37
C	133.20	65.35	2.04	3.92	25.14	Reference
P	66.02	32.24	2.05	1.99	24.86	75.79

**Table 4:** Comparison of MDT: predicted vs. experimental data of the PVA-PDM formulation (*n* ≥ 3; x ± s).

SA/V Ratio/mm^−1^	MDT Prediction/min	MDT Experiment/min	RMSEP/min
0.90	71.70	74.06 ± 11.45	2.36
1.60	33.83	31.04 ± 2.20	2.79
2.30	21.07	16.65 ± 0.46	4.42
4.67	8.36	6.93 ± 0.71	1.43

**Table 5:** Comparison of MDT: predicted vs. experimental data of the EVA-LD formulation (*n* ≥ 3; x ± s).

SA/V Ratio/mm^−1^	MDT Prediction/min	MDT Experiment/min	RMSEP/min
1.73	82.25	78.79 ± 7.24	3.46
1.89	70.74	62.60 ± 5.90	8.14
4.67	15.13	14.40 ± 0.77	0.73

**Table 6:** Comparison of MDT: predicted vs. experimental data of the PVA-PZQ formulation (*n* ≥ 3; x ± s).

SA/V Ratio/mm^−1^	MDT Prediction/min	MDT Experiment/min	RMSEP/min
1.30	54.42	55.91 ± 1.11	1.49
1.83	36.79	38.73 ± 1.07	1.94
2.30	28.32	27.88 ± 2.23	0.44
4.67	12.58	12.16 ± 0.96	0.42

**Table 7:** R^2^ values of model fits.

PVA-PDM Formulation						
SA/V mm^−1^	KMP	Hixson	Peppas Sahlin *n* = 0.79	Hopfenberg	Lapidus + Lordi	Weibull
0.8	0.9837	0.9951	0.9991	0.9837	0.9837	0.1240
1.0	0.9971	0.9969	0.9989	0.9971	0.9971	0.1620
1.5	0.9981	0.9964	0.9995	0.9981	0.9981	0.2575
2.0	0.9966	0.9961	0.9996	0.9966	0.9966	0.9919
2.5	0.9783	0.9975	0.9978	0.9783	0.9783	0.9957
3.3	0.9982	0.9951	0.9997	0.9982	0.9982	0.9955
4.0	0.9949	0.9964	0.9995	0.9949	0.9949	0.9971
5.0	0.9931	0.9987	0.9999	0.9931	0.9931	0.9994
6.0	0.9970	0.9987	0.9999	0.9970	0.9970	0.9997
**PVA-PZQ Formulation**						
**SA/V** **mm^−1^**	**KMP**	**Hixson**	**Peppas Sahlin** ***n*** **= 1.1**	**Hopfenberg**	**Lapidus + Lordi**	**Weibull**
0.8	0.9680	0.9922	0.9980	0.9680	0.9680	0.9993
1.0	0.9870	0.9832	0.9953	0.9870	0.9870	0.9972
1.5	0.9765	0.9727	0.9890	0.9765	0.9765	0.9976
2.0	0.9663	0.9646	0.9667	0.9663	0.9663	0.9973
2.5	0.9819	0.9292	0.9932	0.9819	0.9819	0.9963
3.3	0.9350	0.9408	0.9807	0.9350	0.9350	0.9980
4.0	0.9331	0.9361	0.9877	0.9331	0.9331	0.9975
5.0	0.9143	0.9787	0.9823	0.9143	0.9143	0.9994
6.0	0.9429	0.9299	0.9888	0.9429	0.9429	0.9992
**EVA-LD Formulation**						
**SA/V** **mm^−1^**	**KMP**	**Weibull**	**Peppas Sahlin** ***n*** **= 0.66**	**Higuchi**		
0.9	0.9941	0.9986	0.9808	0.8760		
1.1	0.9910	0.9936	0.9853	0.9304		
1.5	0.9961	0.9961	0.9981	0.9945		
1.7	0.9925	0.9884	0.9966	0.9880		
1.9	0.9949	0.9894	0.9989	0.9941		
2.5	0.9724	0.9979	0.9993	0.9282		
4.0	0.9979	0.9928	0.9934	0.9730		
5.0	0.9970	0.9944	0.9956	0.9583		
6.0	0.9974	0.9946	0.9957	0.9600		

**Table 8:** Calculated constants for the SA/V ratios of both BCS I formulations.

PVA−PDM Formulation					
SA/V	k_1_	k_2_	ln(SA/V)	ln(k_1_)	ln(k_2_)
0.8	1.986	0.010	−0.223	0.686	−4.608
1.0	2.516	0.011	0.000	0.923	−4.549
1.5	3.920	0.027	0.405	1.366	−3.626
2.0	5.561	0.067	0.693	1.716	−2.704
2.5	7.362	0.135	0.916	1.996	−2.001
3.3	11.555	0.258	1.203	2.447	−1.353
4.0	15.459	0.545	1.386	2.738	−0.608
5.0	16.888	0.713	1.609	2.827	−0.338
6.0	20.856	1.069	1.792	3.038	0.066
**EVA−LD Formulation**					
**SA/V**	**k_1_**	**k_2_**	**ln(SA/V)**	**ln(k_1_)**	**ln(k_2_)**
0.9	1.952	0.010	−0.105	0.669	−4.614
1.1	2.286	0.014	0.065	0.827	−4.282
1.5	3.115	0.020	0.405	1.136	−3.928
1.7	4.087	0.031	0.513	1.408	−3.465
1.9	5.009	0.053	0.626	1.611	−2.944
2.5	7.636	0.153	0.916	2.033	−1.876
4.0	10.374	0.279	1.386	2.339	−1.277
5.0	14.444	0.540	1.609	2.670	−0.617
6.0	16.816	0.727	1.792	2.822	−0.319

**Table 9:** Calculated constants for the SA/V ratios of the BCS II–formulation.

PVA-PZQ Formulation					
SA/V	a	b	ln(SA/V)	ln(a)	ln(b)
0.8	105.0	1.38	−0.223	4.654	0.322
1.0	81.0	1.45	0.000	4.394	0.372
1.5	52.0	1.63	0.405	3.951	0.489
2.0	38.0	1.80	0.693	3.638	0.588
2.5	31.5	1.91	0.916	3.450	0.647
3.3	20.8	2.05	1.194	3.035	0.718
4.0	17.1	2.15	1.386	2.836	0.765
5.0	11.5	2.29	1.609	2.442	0.829
6.0	11.2	2.40	1.792	2.414	0.875

## Supplementary Materials

The following are available online at https://www.mdpi.com/article/10.3390/pharmaceutics13091453/s1, Table S1: Physical characterization of the printed tablets for SA/V ratio 1–2 mm^−1^ (*n* ≥ 3, x ± s), Table S2: Physical characterization of the printed tablets for the correlation generation with the PDM-PVA formulation (*n* ≥ 3, x ± s), Table S3: Physical characterization of the printed tablets for the correlation generation with the LD-EVA formulation (*n* ≥ 3, x ± s), Table S4: Physical characterization of the printed tablets for the correlation generation with the PZQ-PVA formulation (*n* ≥ 3, x ± s), Table S5: Physical characterization of the printed tablets for the prediction validation with the PDM-PVA formulation (*n* ≥ 3, x ± s), Table S6: Physical characterization of the printed tablets for the prediction validation with the LD-EVA formulation (*n* ≥ 3, x ± s), Table S7: Physical characterization of the printed tablets for the prediction validation with the PZQ-PVA formulation (*n* ≥ 3, x ± s).
