# Supplementary material for: Predicting Drug Release from 3D Printed Oral Medicines Based on the Surface Area to Volume Ratio of Tablet Geometry
Source: Pharmaceutics. 2021 Sep 11;13(9):1453. doi: 10.3390/pharmaceutics13091453 (PMC8471793; doi:10.3390/pharmaceutics13091453)
Supplement: Supplementary file 1 [file pharmaceutics-13-01453-s001.zip › pharmaceutics-1358929-supplementary.pdf]

pharmaceutics

Supplementary Materials: Predicting Drug Release from 3D Printed Oral Medicines Based on the Surface Area to Volume Ratio of Tablet Geometry

Hellen Windolf, Rebecca Chamberlain and Julian Quodbach *

Institute of Pharmaceutics and Biopharmaceutics, Heinrich Heine University, Universitätsstr. 1, 40225 Düsseldorf, Germany; hellen.windolf@hhu.de (H.W.); rebecca.chamberlain@hhu.de (R.C.)
* Correspondence: julian.quodbach@hhu.de; Tel.: +49-211-81-15693

Table S1. Physical characterization of the printed tablets for SA/V ratio 1 – 2 mm^{-1} (n ≥ 3, x ±s).

PDM	SA/V	mg Total	mg API	h / mm	l / mm	w / mm
Q1	1	706.09 ± 1.07	36.78 ± 0.12	2.61 ± 0.04	15.13± 0.04	15.13± 0.03
Q2	1	306.10 ± 1.06	15.98 ± 0.05	3.98 ± 0.02	8.12 ± 0.07	8.10 ± 0.05
Q3	1	308.52 ± 1.18	16.08 ± 0.02	5.02 ± 0.03	10.14 ± 0.02	5.02 ± 0.06
C	1	241.41 ± 1.55	12.60 ± 0.09	4.01 ± 0.01	7.99 ± 0.01	8.01 ± 0.01
HC	1	805.42 ± 4.61	42.22 ± 0.22	10.01 ± 0.02	11.00 ± 0.02	11.00 ± 0.01
P	1	319.51 ± 0.72	16.67 ± 0.08	9.07 ± 0.05	8.99 ± 0.02	9.04 ± 0.04
**PDM**	**SA/V**	**mg Total**	**mg API**	**h / mm**	**l / mm**	**w / mm**
Q1	1.5	422.56 ± 2.36	22.03 ± 0.06	1.59 ± 0.05	15.16 ± 0.05	15.16 ± 0.05
Q2	1.5	155.65 ± 1.48	8.12 ± 0.09	1.97 ± 0.02	8.10 ± 0.05	8.11 ± 0.04
Q3	1.5	135.64 ± 0.75	7.04 ± 0.06	2.19 ± 0.02	10.14 ± 0.04	5.10 ± 0.00
C	1.5	121.10 ± 0.82	6.27 ± 0.03	1.98 ± 0.02	8.05 ± 0.03	7.99 ± 0.01
HC	1.5	263.57 ± 1.36	13.73 ± 0.09	4.00 ± 0.02	10.03 ± 0.02	10.03 ± 0.01
P	1.5	93.87 ± 0.86	4.94 ± 0.05	5.9 ± 0.00	6.06 ± 0.02	6.07 ± 0.02
**PDM**	**SA/V**	**mg total**	**mg API**	**h / mm**	**l / mm**	**w / mm**
Q1	2	293.76 ± 4.75	15.24 ± 0.22	1.09 ± 0.04	15.13 ± 0.06	15.15 ± 0.04
Q2	2	101.99 ± 1.18	5.29 ± 0.07	1.28 ± 0.02	8.13 ± 0.07	8.13 ± 0.05
Q3	2	86.47 ± 0.76	4.51 ± 0.02	1.4 ± 0.01	10.10 ± 0.03	5.09 ± 0.03
C	2	76.69 ± 0.46	3.97 ± 0.03	1.3 ± 0.01	8.02 ± 0.05	8.04 ± 0.05
HC	2	133.45 ± 0.44	6.96 ± 0.01	1.98 ± 0.02	10.05 ± 0.02	10.00 ± 0.01
P	2	38.75 ± 0.22	2.02 ± 0.01	4.16 ± 0.04	4.44 ± 0.05	4.48 ± 0.11

Table S2. Physical characterization of the printed tablets for the correlation generation with the PDM-PVA formulation (*n* ≥ 3, x ±s).

PDM					
**SA/V / mm^{-1}**	**mg Total**	**mg API**	**h / mm**	**l / mm**	**w / mm**
0.8	464.36 ± 2.40	22.82 ± 0.11	8.01 ± 0.00	8.01 ± 0.01	8.00 ± 0.01
2.5	90.15 ± 3.00	4.54 ± 0.15	4.02 ± 0.01	7.00 ± 0.02	7.00 ± 0.02
3.33	78.46 ± 0.11	4.07 ± 0.02	1.01 ± 0.01	15.01 ± 0.01	15.00 ± 0.01
4	33.40 ± 0.47	1.75 ± 0.03	1.01 ± 0.00	10.00 ± 0.01	10.00 ± 0.00
5	49.1 ± 0.93	2.56 ± 0.05	0.67 ± 0.01	18.02 ± 0.01	18.01 ± 0.01
6	34.87 ± 1.68	1.83 ± 0.09	0.49 ± 0.00	20.00 ± 0.01	20.00 ± 0.01

Table S3. Physical characterization of the printed tablets for the correlation generation with the LD-EVA formulation (*n* ≥ 3, x ±s).

LD					
**SA/V / mm^{-1}**	**mg Total**	**mg API**	**h / mm**	**l / mm**	**w / mm**
0.9	230.80 ± 3.42	22.43 ± 0.36	4.10 ± 0.06	9.90 ± 0.03	9.92 ± 0.05
1.0	174.75 ± 6.33	17.48 ± 0.68	3.01 ± 0.01	10.02 ± 0.02	10.01 ± 0.01
1.5	204.33 ± 11.36	22.18 ± 1.24	3.96 ± 0.01	9.01 ± 0.06	9.02 ± 0.06
1.87	349.59 ± 0.72	37.68 ± 0.19	10.05 ± 0.04	10.01 ± 0.03	10.00 ± 0.01
2.5	67.25 ± 4.05	7.25 ± 0.42	4.11 ± 0.02	6.83 ± 0.02	6.86 ± 0.04

4	31.50 ± 0.42	3.71 ± 0.04	0. 96 ± 0.12	10.70 ± 0.67	10.70 ± 0.77
5	32.44 ± 1.85	3.83 ± 0.23	0.50 ± 0.00	13.97 ± 0.35	14.11 ± 0.12
6	25.67 ± 1.67	3.02 ± 0.14	0.50 ± 0.00	17.93 ± 0.06	17.90 ± 0.10

Table S4. Physical characterization of the printed tablets for the correlation generation with the PZQ-PVA formulation (*n* ≥ 3, x ±s).

PZQ-PVA					
SA/V / mm^{-1}	mg Total	mg API	h / mm	l / mm	w / mm
0.8	308.21 ± 11.73	14.85 ± 1,05	5.00 ± 0.08	10.22 ± 0.21	10.06 ± 0.04
1	810.24 ± 16.81	32.32 ± 1.25	4.01 ± 0.04	8.40 ± 0.10	8.42 ± 0.08
1.5	224.11 ± 17.60	11.03 ± 0.84	4.05 ± 0.07	10.11 ± 0.07	10.13 ± 0.03
2	118.123 ± 7.67	5.56 ± 0.34	2.02 ± 0.02	10.04 ± 0.05	10.04 ± 0.04
2.5	90.38 ± 3.76	4.47 ± 0.22	3.96 ± 0.04	7.20 ± 0.15	7.19 ± 0.07
3.33	46.08 ± 1.15	2.11 ± 0.05	1.02 ± 0.03	15.01 ± 0.01	14.97 ± 0.11
4	33.04 ± 0.54	1.37 ± 0.04	1.00 ± 0.03	10.02 ± 0.08	10.02 ± 0.07
5	45.46 ± 0.17	1.97 ± 0.03	0.59 ± 0.05	17.98 ± 0.06	17.99 ± 0.04
6	32.05 ± 0.27	1.11 ± 0.07	0.58 ± 0.03	19.97 ± 0.12	19.95 ± 0.04

Table S5. Physical characterization of the printed tablets for the prediction validation with the PDM-PVA formulation (*n* ≥ 3, x ±s).

PDM-PVA					
SA/V / mm^{-1}	mg Total	mg API	h / mm	l / mm	w / mm
0.9	294.00 ± 3.48	14.83 ± 0.20	4.90 ± 0.02	8.02 ± 0.01	8.00 ± 0.02
1.6	163.14 ± 1.49	8.33 ± 0.07	2.41 ± 0.03	10.02 ± 0.03	9.99 ± 0.04
2.3	103.20 ± 1.46	5.19 ± 0.06	2.00 ± 0.01	10.00 ± 0.01	10.01 ± 0.01
4.67	39.54 ± 2.15	2.15 ± 0.14	0.59 ± 0.02	12.02 ± 0.04	12.03 ± 0.05

Table S6. Physical characterization of the printed tablets for the prediction validation with the LD-EVA formulation (*n* ≥ 3, x ±s).

LD-EVA					
SA/V / mm^{-1}	mg Total	mg API	h / mm	l / mm	w / mm
1.73	210.63 ± 6.46	22.71± 0.88	4.73 ± 0.21	10.12 ± 0.16	10.13 ± 0.18
1.89	329.30 ± 5.04	34.53 ± 0.39	4.17 ± 0.06	11.83 ± 0.15	12.62 ± 0.37
4.67	24.46 ± 0.41	2.93 ± 0.11	0.61 ± 0.02	11.98 ± 0.08	12.07 ± 0.16

Table S7. Physical characterization of the printed tablets for the prediction validation with the PZQ-PVA formulation (*n* ≥ 3, x ±s).

PZQ-PVA					
SA/V / mm^{-1}	mg Total	mg API	h / mm	l / mm	w / mm
1.3	281.393 ± 1.36	14.86 ± 0.09	6.96 ± 0.11	11.01 ± 0.08	11.02 ± 0.06
1.83	186.42 ± 2.83	9.64 ± 0.20	4.01 ± 0.06	10.17 ± 0.07	10.14 ± 0.05
2.3	100.98 ± 1.05	5.11 ± 0.04	1.99 ± 0.05	10.21 ± 0.01	10.20 ± 0.04
4.67	41.92 ± 0.77	1.72 ± 0.03	0.59 ± 0.01	12.36 ± 0.13	12.39 ± 0.15
